# Retraction: Long noncoding RNA TUG1 promotes proliferation and inhibits apoptosis in multiple myeloma by inhibiting miR-29b-3p

**DOI:** 10.1042/BSR-2018-2489_RET

**Published:** 2021-06-21

**Keywords:** miR-29b-3p, multiple myeloma, TUG1

This article is being retracted from *Bioscience Reports* at the request of the authors following receipt of a notification from a reader, alerting the authors to the duplication of Figure 2C, which appears in serval other publications by different authors.

The authors wish to retract the article. The Editor-in-Chief and Editorial Board agree with the Retraction.

